# Paternal childcare at 6 months and risk of maternal psychological distress at 1 year after delivery: The Japan Environment and Children’s Study (JECS)

**DOI:** 10.1192/j.eurpsy.2021.2213

**Published:** 2021-06-09

**Authors:** Haruka Kasamatsu, Akiko Tsuchida, Kenta Matsumura, Kei Hamazaki, Hidekuni Inadera

**Affiliations:** 1 Toyama Regional Center for JECS, University of Toyama, Toyama, Japan; 2 Department of Public Health, Faculty of Medicine, University of Toyama, Toyama, Japan; Present affiliation: Kei Hamazaki, Department of Public Health, Graduate school of Medicine, Gunma University, Maebashi, Japan.

**Keywords:** Birth cohort, caregiving, father, maternal mental health, play

## Abstract

**Background:**

Paternal childcare is reported to benefit maternal mental health, but specific childcare behaviors have not been comprehensively determined. This study sought to identify paternal childcare behaviors associated with maternal mental health by adjusting for other covariates associated with maternal mental health and examining childcare behaviors.

**Methods:**

This study investigated whether seven types of paternal childcare behaviors at 6 months after delivery were associated with maternal psychological distress at 1 year after delivery, which was assessed using the Kessler Psychological Distress Scale (K6). After exclusions from a dataset of 103,062 pregnancies obtained in the Japan Environment and Children’s Study, we evaluated data from 75,607 mothers.

**Results:**

More than 70% of fathers were always or sometimes involved in “playing at home,” “playing outdoors,” “changing diapers,” and “bathing,” 60%–70% in “helping with feeding” and “dressing,” and 45.9% in “putting the child to bed.” All paternal childcare behaviors showed some beneficial association with less maternal psychological distress, both moderate (K6 score 5–12) and severe (K6 score ≥ 13) distress. Playing at home was the most beneficial association identified (adjusted odds ratio [aOR] 0.66, 95% confidence interval [CI] 0.56–0.76 for moderate psychological distress; aOR 0.36, 95% CI 0.28–0.48 for severe psychological distress).

**Conclusions:**

These seven types of paternal childcare behaviors may help lessen maternal psychological distress. Emphasis should be given to building education systems and working environments that promote paternal childcare.

## Introduction

Maintenance and improvement of maternal mental health is reported to be as a pressing issue in the field of maternal and child health. In the general Japanese population, the prevalence of mood disorders and anxiety disorders is 3.1% and 4.8%, respectively [1], indicating that many Japanese people have poor mental health. Among mothers, poor mental health not only diminishes their quality of life, but has also been shown to negatively affect their children. Examples of negative effects include long-term problems in the mother–child relationship [2–4] and mothers performing fewer suitable childcare behaviors [5]. It has also been suggested that poor maternal mental health can be detrimental children’s health and development [6–8]. Thus, maintenance and improvement of maternal mental health are paramount for children’s healthy growth.

Poor maternal mental health is reported to be associated with a myriad of factors including history of previous depression, prenatal anxiety, single marital status, and low socioeconomic status [9]. The support of close family members is also related to maternal mental health [10,11] with the support of partners playing a particularly important role [12,13]. Some studies have examined the association of less paternal time engaged in childcare or child-related chores with maternal depression, anxiety, and stress [14–16]. According to the World Gender Gap Report, Japan ranked 121st out of 153 countries in the Global Gender Gap Index 2020 [17]. Among East Asian countries, which historically have a patriarchal culture, Japan has one of the largest gender gaps in participation in political and economic activities. Japan’s Ministry of Internal Affairs and Communications has reported that the time spent by mothers in childcare for children under the age of 6 years is 4.7 times that spent by fathers [18]. Moreover, Japanese fathers spend on average 41 min a day engaged in unpaid work at home, including housework and childcare, which is far less than the average of 136 min in The Organisation for Economic Co-operation and Development (OECD) countries [19]. To address this, the Ministry of Health, Labour and Welfare’s Healthy Parents and Children 21 campaign has set concrete numerical goals for maternal and child health, one of which is to promote paternal childcare [20].

In countries with large gender gaps such as Japan, where the time that fathers spend engaged in childcare is low, it is important to know which of their behaviors are associated with improved mental health of the mothers, in addition to spending more time engaged in childrearing activities. However, no studies have examined the association between paternal childcare behaviors and mothers’ mental health. If the specific paternal childcare behaviors that are beneficially associated with mothers’ mental health can be identified, then specific recommendations can be given for fathers. Some challenges exist in determining such associations. For example, longitudinal studies face the problem of maintaining and examining a sufficient sample size. Thus, assessment of the association between paternal childcare behaviors and maternal mental health requires adjusting for these other factors. To address this, in this study, we analyzed data from the Japan Environment and Children’s Study (JECS), a large-scale cohort study, to determine the frequency of some specific paternal childcare behaviors in a general Japanese population in order to examine how such behaviors are associated with maternal mental health.

## Methods

### Study design

The JECS is a government-funded, nationwide birth cohort study that aims to evaluate the impact of environmental factors on children’s health and development. In total, 103,062 pregnancies were registered from 15 Regional Centers across Japan between 2011 and 2014, and detailed descriptions of the JECS can be found elsewhere [21,22]. Participant recruitment involved a face-to-face explanation of the survey to mothers, and written informed consent was obtained and recorded. The authors assert that all procedures contributing to the present work comply with the ethical standards of the relevant national and institutional committees on human experimentation and with the Helsinki Declaration of 1975, as revised in 2008. All procedures involving human subjects in the JECS protocol were approved by the Institutional Review Board on Epidemiological Studies of the Ministry of the Environment (100910001), and the ethics committees of all participating institutions.

### Study data

**Figure 1.**
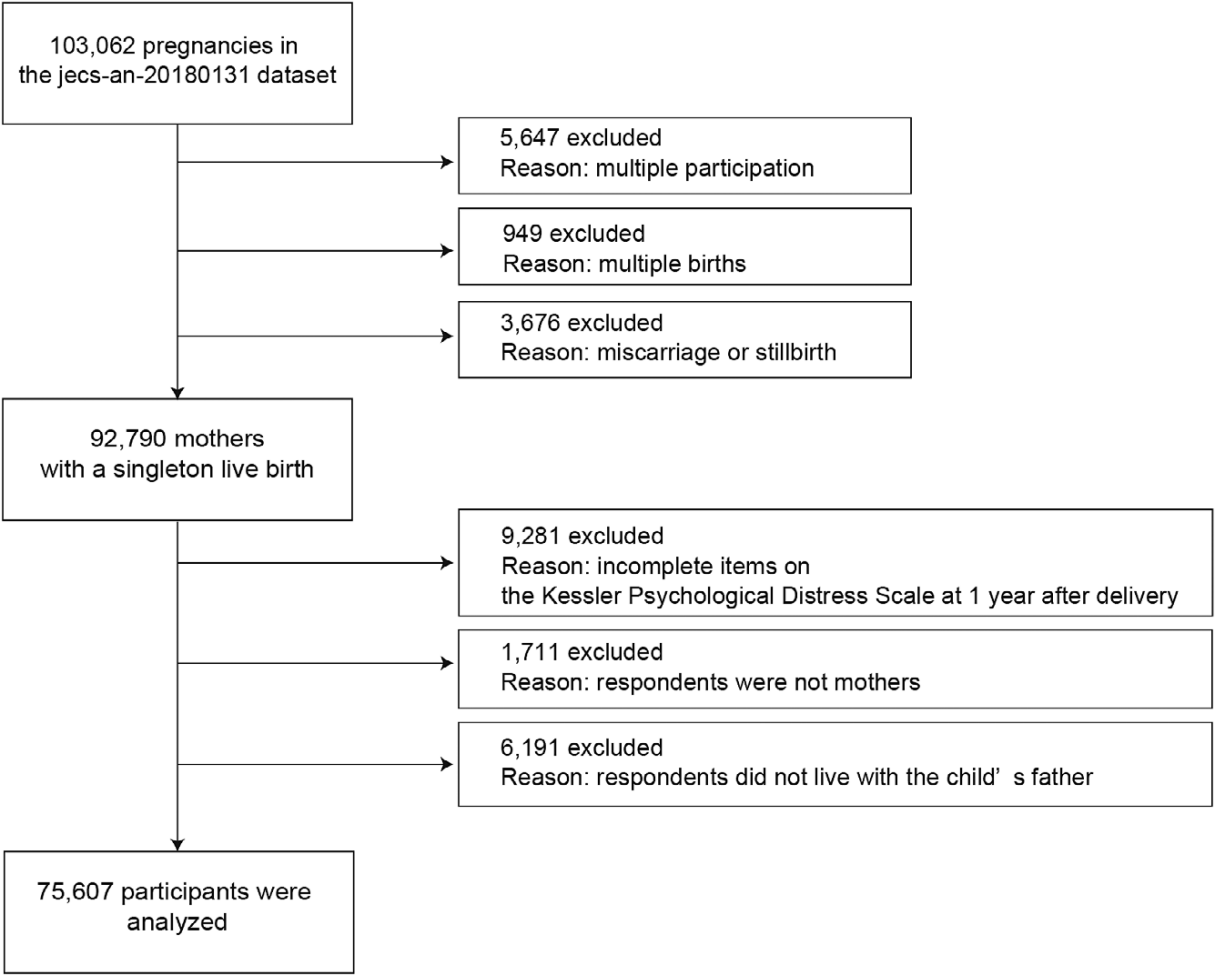
Participant flow diagram.

This study examined data from 92,790 mothers with singleton, live births recorded in a JECS dataset (jecs-an-20180131); data from mothers with multiple participations (5,647), multiple births (949), and stillbirths/miscarriages (3,676) were excluded. After eliminating cases with incomplete items on the Kessler Psychological Distress Scale (K6) at 1 year after delivery and mothers who did not live with the child’s father (i.e., the mother’s partner), data from 75,607 mothers were analyzed (Figure 1). Of these mothers, 98.9% responded that they were married.

### Measurements

The self-report questionnaire was administered to the mothers at registration, during the second/third trimester, and again at 1 month, 6 months, and 1 year after delivery. Data were collected on demographics, medical and obstetric history, physical and mental health issues, lifestyle factors, occupation, and socioeconomic status.

### Independent variables

Based on the mothers’ survey responses at 6 months after delivery, paternal childcare was assessed for the following seven behaviors: (a) playing at home, (b) playing outdoors, (c) helping with feeding, (d) changing diapers, (e) dressing, (f) bathing, and (g) putting the child to bed. Responses were provided on a 4-point scale (always, sometimes, rarely, and not at all). We selected these behaviors because all have been similarly assessed in previous studies conducted in Western countries, in other Asian countries, and in Japan [12,23–27].

### Dependent variables

Maternal mental health was assessed in terms of psychological distress, using the K6, at 1 year after delivery. The K6 was developed by Kessler et al. [28], and the validity of the Japanese version was determined by Furukawa et al. [29]. The six items are assessed on a 5-point scale (0–4), yielding a total score of 0–24. Psychological distress was assessed over the past 4 weeks, with a higher score representing greater psychological distress. Severe mental illness is defined as a K6 score ≥ 13, a score proposed by Kessler et al. [28] as the optimal cut-off for achieving a balance between false positive and false negative results. This cut-off has been widely used in epidemiological studies [30]. The optimal cut-off for the Japanese version was determined to be 4/5 by Sakurai et al. [31], and thus a K6 score ≥ 5 has been proposed as the optimal cut-off to be comparable with the Center for Epidemiologic Studies Depression Scale in the detection of mood and anxiety disorders. The present study operationally defined K6 scores of ≥13 and 5–12 as severe psychological distress and moderate psychological distress, respectively. Cronbach’s alpha was 0.85 at both 6 months and 1 year after delivery.

### Covariates

Potential confounding factors and covariates were included in the statistical analysis if they were found in previous studies using JECS data [3,4,32,33] to be associated (or are theoretically inferred to be associated) with the outcome. The covariates comprised the following variables: maternal age at 1 month after birth (≤24 years, 25–29 years, 30–34 years, or ≥35 years), parity (nullipara or multipara), educational background (junior high school or high school; technical junior college, technical/vocational college or associate degree; or bachelor’s degree, postgraduate degree), annual household income (<4 million, 4 to <6 million, or ≥6 million JPY), alcohol intake (never drinker, ex-drinker, or current drinker), smoking status (never smoker, ex-smoker, or current smoker), passive smoking (no, outdoors, or yes), employment status (no job, self-employed, permanent full-time employee, part-time or temporary employee, or other), history of depression (yes or no), history of anxiety disorder (yes or no), history of schizophrenia (yes or no), history of dysautonomia (yes or no), diagnostic record of mental disorder during pregnancy (yes or no), psychological distress during pregnancy (total K6 score ≤4, 5–12, or ≥13), postpartum depression at 1 month after delivery (yes or no), experiencing sadness during the past year (yes or no), anomaly in the infant (yes or no), infant’s sex (male or female), intensity and frequency of infants’ crying (quite often and long, sometimes and short, or hardly), co-resident family members during pregnancy (parent(s) or parent(s)-in-law), partner’s age at 6 months after delivery (≤24 years, 25–29 years, 30–34 years, or ≥35 years), partner’s highest educational level (junior high school or high school; technical junior college, technical/vocational college, or associate degree; or bachelor’s degree, postgraduate degree). This study defined anomaly in the infant as one or more of 31 congenital anomalies that are easily detectable at delivery and that generally require prompt medical attention after delivery [34]. Postpartum depression at 1 month after delivery was assessed using the Edinburgh Postnatal Depression Scale (EPDS), with a score of ≥9 used as a cut-off [35,36] to categorize results. Cronbach’s alpha for the EPDS was 0.82, as shown in our previous study [37].

### Statistical analysis

To estimate the risks of severe psychological distress and moderate psychological distress for each level of paternal childcare, we performed multinomial logistic regression analysis to calculate odds ratios (ORs) and 95% confidence intervals (CIs). Two-sided *p* values less than 0.05 were considered to indicate statistical significance. Missing data of covariates were included as categorical variables in the adjusted model. Data were analyzed using SAS version 9.4 software (SAS Institute Inc., Cary, NC).

## Results

Table 1 shows the participants’ characteristics. More than 60% of the mothers were aged ≥30 years and more than half were multipara. Overall, 45.3% of mothers responded that they were full-time homemakers or were unemployed while pregnant. During pregnancy, 24.4% of mothers were classified as having moderate psychological distress and 2.7% as having severe psychological distress; 13.2% demonstrated symptoms of postpartum depression. Approximately 60% of mothers responded that their annual household income was ≥4 million JPY. Overall, 5.9% of mothers lived with their parents and 12.2% lived with their parents-in-law. There were slightly more infant boys (51.3%) than girls (48.7%). The most common age range of partners was ≥35 years (43.0%).

**Table 1.** Participants’ demographics and obstetric characteristics (*N* = 75,607).

Characteristics	*N* (%)
Maternal age (years) at 1 month after delivery, *n* (%)	
≤24	5,132 (6.8)
25–29	20,134 (26.6)
30–34	27,901 (36.9)
≥35	22,121 (29.3)
Missing	319 (0.4)
Previous delivery, *n* (%)	
Nullipara	31,839 (42.1)
Multipara	43,763 (57.9)
Missing	5 (0.0)
Highest educational level, *n* (%)	
Junior high school or high school	24,962 (33.0)
Technical junior college, technical/vocational college or associate degree	32,510 (43.0)
Bachelor’s degree, postgraduate degree	17,424 (23.1)
Missing	711 (0.9)
Annual household income (JPY), *n* (%)	
<4 million	26,734 (35.4)
4 to <6 million	24,107 (31.9)
≥6 million	19,792 (26.2)
Missing	4,974 (6.6)
Alcohol intake at 1 month after delivery, *n* (%)	
Never drinker	68,980 (91.2)
Ex-drinker	3,231 (4.3)
Current drinker	2,905 (3.8)
Missing	491 (0.7)
Smoking status at 1 month after delivery, *n* (%)	
Never smoker	45,491 (60.2)
Ex-smoker	27,099 (35.8)
Current smoker	2,461 (3.3)
Missing	556 (0.7)
Passive smoking, *n* (%)	
No	37,281 (49.3)
Outdoors	36,209 (47.9)
Yes	1,624 (2.2)
Missing	493 (0.7)
Employment status, *n* (%)	
Unemployed	34,263 (45.3)
Permanent full-time employee	23,488 (31.1)
Part-time or temporary employee	12,982 (17.2)
Self-employed	2,630 (3.5)
Other	1,353 (1.8)
Missing	891 (1.2)
History of depression, yes (%)	2,190 (2.9)
Missing	0 (0.0)
History of anxiety disorder, yes (%)	2,025 (2.7)
Missing	0 (0.0)
History of schizophrenia, yes (%)	115 (0.2)
Missing	0 (0.0)
History of dysautonomia, yes (%)	2,712 (3.6)
Missing	0 (0.0)
Diagnostic record of mental disorder during pregnancy, yes (%)	522 (0.7)
Missing	0 (0.0)
Psychological distress during pregnancy (total K6 score), *n* (%)	
≤4	54,567 (72.2)
5–12	18,442 (24.4)
≥13	2,068 (2.7)
Missing	530 (0.7)
Postpartum depression at 1 month after delivery^a^, yes (%)	9,951 (13.2)
Missing	1,315 (1.7)
Experiencing sadness during the past year, yes (%)	31,662 (41.9)
Missing	2,878 (3.8)
Anomaly in the infant, yes (%)	1,702 (2.3)
Missing	0 (0.0)
Infant’s sex, *n* (%)	
Male	38,779 (51.3)
Female	36,828 (48.7)
Missing	0 (0.0)
Intensity and frequency of infant’s crying, *n* (%)	
Quite often and long	12,654 (16.7)
Sometimes and short	58,824 (77.8)
Hardly ever	3,381 (4.5)
Missing	748 (1.0)
Living with	
Parent(s), yes (%)	4,435 (5.9)
Parent(s)-in-law, yes (%)	9,243 (12.2)
Missing	0 (0.0)
Partner’s age (years) at 6 months after delivery, n (%)	
≤24	2,673 (3.5)
25–29	13,986 (18.5)
30–34	24,959 (33.0)
≥35	32,539 (43.0)
Missing	1,450 (1.9)
Partner’s highest educational level, *n* (%)	
Junior high school or high school	31,226 (41.3)
Technical junior college, technical/vocational college, or associate degree	17,223 (22.8)
Bachelor’s degree, postgraduate degree	26,294 (34.8)
Missing	864 (1.1)

a Postpartum depression: total EPDS score of ≥9.

Table 2 shows paternal involvement in each aspect of childcare. More than 70% of mothers responded that fathers always or sometimes played at home and played outdoors with their child. Play at home was the most frequent of the seven behaviors, with 47.4% of mothers responding that their partners always played with their child at home. Among the caregiving behaviors, bathing was the most frequent, with 41.1% of mothers responding the father always bathed the child, and 60–70% of fathers always or sometimes performed the other caregiving behaviors, except for putting the child to bed, which was the least frequent among the seven behaviors (45.9% of fathers always or sometimes involved).

**Table 2.** Frequency of paternal involvement in different childcare behaviors.

	Response items (*n*, %)			
	Not at all	Rarely	Sometimes	Always
Play				
Playing at home	1,124 (1.5)	4,264 (5.7)	34,137 (45.5)	35,545 (47.4)
Playing outdoors	8,089 (10.8)	13,807 (18.4)	38,326 (51.1)	14,826 (19.8)
Caregiving				
Helping with feeding	13,568 (18.2)	14,612 (19.5)	30,931 (41.4)	15,659 (20.9)
Changing diapers	7,593 (10.1)	12,336 (16.4)	38,217 (50.9)	16,948 (22.6)
Dressing	8,781 (11.7)	14,375 (19.1)	36,575 (48.7)	15,361 (20.5)
Bathing	7,058 (9.4)	7,380 (9.8)	29,817 (39.7)	30,826 (41.1)
Putting the child to bed	19,673 (26.2)	20,950 (27.9)	26,281 (35.0)	8,152 (10.9)

Table 3 shows the ORs for all paternal childcare behaviors in relation to moderate maternal psychological distress (K6 score 5–12) and severe maternal psychological distress (K6 score ≥ 13) at 1 year after delivery. Overall, even after adjustments were made for all covariates, with “not at all” used as a reference, all paternal childcare behaviors showed some significant associations with both moderate and severe maternal psychological distress. The ORs for severe psychological distress were generally lower than those for moderate psychological distress. For both moderate and severe psychological distress, the ORs for play behaviors were roughly equal to or lower than the ORs for caregiving behaviors. The following trends were observed for the two play behaviors. When fathers always or sometimes played with their child at home, fewer mothers tended to have moderate or severe psychological distress at 1 year after delivery. Trends were generally similar for fathers who played with their child outdoors. The following trends were observed in the five caregiving behaviors. When fathers always or sometimes helped with feeding, changing diapers, dressing, and bathing their child, fewer mothers tended to have moderate or severe psychological distress at 1 year after delivery. When fathers always or sometimes put the child to bed, fewer mothers tended to have moderate psychological distress. When fathers sometimes or rarely put the child to bed, this tended to be beneficial only for mothers with severe psychological distress.

**Table 3.** Maternal psychological distress at 1 year after delivery and paternal childcare involvement at 6 months after delivery.

Variable	Moderate psychological distress		Severe psychological distress	
	K6 scores 5–12		K6 scores ≥ 13	
	Prevalence, case/total (%)	Adjusted OR (95% CI)^a^	Prevalence, case/total (%)	Adjusted OR (95% CI)^a^
*Playing*				
Playing at home				
Not at all	311/1,124 (27.7)	1.00	87/1,124 (7.7)	1.00
Rarely	1,161/4,264 (27.2)	1.05 (0.89–1.24)	227/4,264 (5.3)	0.84 (0.62–1.14)
Sometimes	7,041/34,137 (20.6)	**0.82 (0.71**–**0.95)**	883/34,137 (2.6)	**0.51 (0.39**–**0.67)**
Always	5,889/35,545 (16.6)	**0.66 (0.56**–**0.76)**	635/35,545 (1.8)	**0.36 (0.28**–**0.48)**
Playing outdoors				
Not at all	1,884/8,089 (23.3)	1.00	356/8,089 (4.4)	1.00
Rarely	2,991/13,807 (21.7)	0.94 (0.88–1.02)	407/13,807 (3.0)	**0.75 (0.64**–**0.88)**
Sometimes	7,094/38,326 (18.5)	**0.81 (0.76**–**0.87)**	754/38,326 (2.0)	**0.54 (0.47**–**0.62)**
Always	2,429/14,826 (16.4)	**0.70 (0.65**–**0.75)**	314/14,826 (2.1)	**0.52 (0.44**–**0.62)**
*Caregiving*				
Helping with feeding				
Not at all	2,843/13,568 (21.0)	1.00	411/13,568 (3.0)	1.00
Rarely	3,079/14,612 (21.1)	1.04 (0.97–1.10)	385/14,612 (2.6)	0.96 (0.82–1.12)
Sometimes	5,741/30,931 (18.6)	**0.91 (0.86**–**0.96)**	667/30,931 (2.2)	**0.79 (0.69**–**0.90)**
Always	2,678/15,659 (17.1)	**0.80 (0.75**–**0.86)**	369/15,659 (2.4)	**0.76 (0.65**–**0.89)**
Changing diapers				
Not at all	1,610/7,593 (21.2)	1.00	281/7,593 (3.7)	1.00
Rarely	2,665/12,336 (21.6)	1.06 (0.98–1.15)	356/12,336 (2.9)	0.88 (0.74–1.05)
Sometimes	7,162/38,217 (18.7)	**0.93 (0.87**–**1.00)**	789/38,217 (2.1)	**0.70 (0.60**–**0.81)**
Always	2,969/16,948 (17.5)	**0.84 (0.78**–**0.91)**	408/16,948 (2.4)	**0.73 (0.61**–**0.86)**
Dressing				
Not at all	2,002/8,781 (22.8)	1.00	368/8,781 (4.2)	1.00
Rarely	3,051/14,375 (21.2)	0.95 (0.89–1.02)	381/14,375 (2.7)	**0.72 (0.61**–**0.84)**
Sometimes	6,729/36,575 (18.4)	**0.85 (0.80**–**0.91)**	721/36,575 (2.0)	**0.60 (0.52**–**0.69)**
Always	2,619/15,361 (17.1)	**0.75 (0.70**–**0.80)**	363/15,361 (2.4)	**0.62 (0.53**–**0.73)**
Bathing				
Not at all	1,527/7,058 (21.6)	1.00	249/7,058 (3.5)	1.00
Rarely	1,609/7,380 (21.8)	1.00 (0.91–1.09)	206/7,380 (2.8)	**0.80 (0.65**–**0.99)**
Sometimes	5,723/29,817 (19.2)	**0.91 (0.85**–**0.98)**	665/29,817 (2.2)	**0.73 (0.62**–**0.86)**
Always	5,544/30,826 (18.0)	**0.85 (0.79**–**0.91)**	714/30,826 (2.3)	**0.71 (0.60**–**0.83)**
Putting the child to bed				
Not at all	4,064/19,673 (20.7)	1.00	608/19,673 (3.1)	1.00
Rarely	4,085/20,950 (19.5)	0.99 (0.94–1.04)	454/20,950 (2.2)	**0.79 (0.69**–**0.90)**
Sometimes	4,706/26,281 (17.9)	**0.89 (0.84**–**0.93)**	523/26,281 (2.0)	**0.70 (0.61**–**0.79)**
Always	1,544/8,152 (18.9)	**0.90 (0.84**–**0.97)**	248/8,152 (3.0)	0.91 (0.77–1.08)

a Covariates were adjusted for mother’s and father’s age, parity, mother’s and father’s educational background, annual household income, alcohol intake, smoking status, passive smoking, employment status, history of depression, history of anxiety disorder, history of schizophrenia, history of dysautonomia, diagnostic record of mental disorder during pregnancy, psychological distress during pregnancy, postpartum depression at 1 month after delivery, experiencing sadness during the past year, anomaly in the infant, infant’s sex, intensity and frequency of infants’ crying, co-resident family members during pregnancy (parent(s), or parent(s)-in-law).

Bold type indicates statistical significance (P < 0.05).

Abbreviations: CI, confidence interval; OR, odds ratio.

## Discussion

This study examined how specific paternal childcare behaviors are associated with maternal psychological distress at 1 year after delivery in a general Japanese population and found that all of the paternal childcare behaviors showed some associations with reduced risk of maternal psychological distress. To the best of our knowledge, this is the first prospective study on the association between paternal childcare behaviors for infants and maternal psychological distress in Japan. The results were adjusted for 22 covariates, including maternal mental health history, maternal psychological distress during pregnancy, and depressive symptoms at 1 month postpartum. This suggests that paternal childcare behaviors are associated factors with respect to the maternal psychological distress at 1 year after delivery.

Our finding that paternal childcare is associated with reduced risk of maternal mental health is consistent with that of previous studies conducted with general populations in Western countries and some Asian countries [11,13,15,38]. These studies have shown that women with depression perceive greater inequality in marital decision-making and in the division of household chores compared with women who are not depressed [39]. Accordingly, paternal play and caregiving might lessen maternal feelings of inequality in parenting behavior. Nomaguchi et al. [40] reported that although mothers enjoy spending time with their children, the need to spend long periods of time with their children puts pressure on them, which affects their well-being. Of particular note, our results show that fathers always playing with their children had the lowest OR compared with other caregiving behaviors. Therefore, fathers playing with and watching over their children may relieve some of the pressure on mothers, allowing mothers to have some time to themselves. More than 70% of fathers in our sample always or sometimes played with their children. This can be important for fathers to know, especially if they have little experience with childcare, lack sufficient childcare skills, or work long hours that prevent them from spending enough time interacting with their children.

Almost all paternal childcare behaviors were associated with better maternal mental health; however, for mothers with severe psychological distress, no improvement in mental health was observed when the father always put the child to bed. Although it is not clear why fathers always putting the child to bed differed from other behaviors, this was the least common answer (10.9%) among the behaviors examined (Table 2). Mothers in Japan generally sleep in the same bed with their children, and they can rest at that time. Therefore, the reason why this behavior differed from the other behaviors may be because it was not related to giving the mother time to rest. It is thus necessary to further examine why this behavior yielded different results from the other behaviors.

There are two potential public health implications of our findings. First, this study provides a new perspective to assessing maternal mental health, that is, the degree to which fathers assist with childcare. The addition of this perspective might help with further identification of at-risk populations. If assessment shows that paternal childcare will be difficult to achieve in certain cases, then maternal mental health should be maintained by early implementation of alternative measures such as community or formal support. Second, this study indicates that fathers as well as mothers should be involved in maintaining and improving maternal mental health. For example, starting while the mother is still pregnant, the father can be given opportunities to learn childcare skills (e.g., helping with feeding, changing diapers, and dressing) with the knowledge that his involvement in childcare is helping the mother as well. In addition, it will be important to build working environments that promote paternal childcare. Including fathers offers a greater range of preventive measures against the development of maternal mental health problems.

### Strengths and limitations

This study has two strengths. First, the findings are based on data from a large-scale cohort study that enrolled roughly 100,000 Japanese people and are therefore highly reliable thanks to adjustment for other factors associated with maternal mental health. Second, associations between paternal childcare and maternal mental health were assessed over time, with independent and dependent variables assessed at 6 months and 1 year after delivery, respectively.

This study also has some limitations. First, assessment of paternal childcare behaviors was based not on objective indicators but on subjective assessment (by the mother). According to one study, fathers tend to overestimate their own involvement in childcare and mothers tend to underestimate their involvement [41]. Moreover, mothers perceive support as low when their health status is worse than usual [38]. Future studies could assess the association between paternal childcare and maternal mental health using potentially more reliable methods, such as reviewing videos of paternal behavior. Second, assessments of psychological distress were based on mothers’ self-reports. Mothers with more severe psychological distress may have not been able to respond to the self-report questionnaire or could have been lost to follow-up. Given that psychological distress during pregnancy is a factor related to participants dropping out of the JECS [42], this selection bias must be kept in mind when interpreting the results of the present study. Third, although fathers’ psychological distress and partner relationship quality were also considered to be related to the mother’s psychological distress and parental childcare, these variables could not be analyzed in the present study due to insufficient data or because they were not measured. Lastly, our study involved a general Japanese population and therefore the findings cannot easily be generalized to populations with different sociocultural characteristics or populations of social minorities. Studies of general populations in the West and elsewhere in Asia have also found that paternal childcare protects mothers from psychological distress [11,13,15,38], and although the results of the present study appear to corroborate such findings, views on paternal childcare are reported to be affected by sociocultural background [43]. In addition, for mothers in the United States who were born elsewhere (i.e., a social minority population), support from family and friends is reported to be more effective than paternal support for decreasing childcare stress [44]. Further research is needed to accumulate findings on the effects of paternal childcare involvement on maternal mental health in populations with social backgrounds that differ from that of the population in the present study and in populations of social minorities.

## Conclusion

Highly frequent paternal childcare behaviors were found to be associated with less maternal psychological distress in this analysis of data from a large-scale cohort study that assessed independent and dependent variables at 6 months and 1 year after delivery, respectively. All paternal childcare behaviors (playing at home, playing outdoors, helping with feeding, changing diapers, dressing, bathing, and putting the child to bed) were shown to be associated with less maternal psychological distress. In the future, intervention studies that can bring about behavioral changes in fathers should be conducted to confirm our findings.

## Data Availability

Data are unsuitable for public deposition due to ethical restrictions and legal framework of Japan. It is prohibited by the Act on the Protection of Personal Information (Act No. 57 of 30 May 2003, amendment on 9 September 2015) to publicly deposit the data containing personal information. Ethical Guidelines for Epidemiological Research enforced by the Japan Ministry of Education, Culture, Sports, Science and Technology and the Ministry of Health, Labour and Welfare also restricts the open sharing of the epidemiologic data. All inquiries about access to data should be sent to: jecs-en@nies.go.jp. The person responsible for handling enquiries sent to this e-mail address is Dr. Shoji F. Nakayama, JECS Programme Office, National Institute for Environmental Studies.
